# The *Caenorhabditis elegans* Eph Receptor Activates NCK and N-WASP, and Inhibits Ena/VASP to Regulate Growth Cone Dynamics during Axon Guidance

**DOI:** 10.1371/journal.pgen.1002513

**Published:** 2012-02-23

**Authors:** Ahmed M. Mohamed, Jeffrey R. Boudreau, Fabian P. S. Yu, Jun Liu, Ian D. Chin-Sang

**Affiliations:** Department of Biology, Queen's University, Kingston, Canada; Harvard University, United States of America

## Abstract

The Eph receptor tyrosine kinases (RTKs) are regulators of cell migration and axon guidance. However, our understanding of the molecular mechanisms by which Eph RTKs regulate these processes is still incomplete. To understand how Eph receptors regulate axon guidance in *Caenorhabditis elegans*, we screened for suppressors of axon guidance defects caused by a hyperactive VAB-1/Eph RTK. We identified NCK-1 and WSP-1/N-WASP as downstream effectors of VAB-1. Furthermore, VAB-1, NCK-1, and WSP-1 can form a complex *in vitro*. We also report that NCK-1 can physically bind UNC-34/Enabled (Ena), and suggest that VAB-1 inhibits the NCK-1/UNC-34 complex and negatively regulates UNC-34. Our results provide a model of the molecular events that allow the VAB-1 RTK to regulate actin dynamics for axon guidance. We suggest that VAB-1/Eph RTK can stop axonal outgrowth by inhibiting filopodia formation at the growth cone by activating Arp2/3 through a VAB-1/NCK-1/WSP-1 complex and by inhibiting UNC-34/Ena activity.

## Author Summary

The correct wiring of the nervous system depends on the ability of axons to properly interpret extracellular cues that guide them to their targets. The Eph receptor tyrosine kinases (RTKs) have roles in guiding axons, but their signaling pathways are not completely understood. In this study, we used the nematode *Caenorhabditis elegans* to study how the VAB-1 Eph RTK regulates the growth cone structure for axon guidance. Genetic and molecular data show that VAB-1 regulates the conserved molecules NCK-1, WSP-1/N-WASP, and UNC-34/Ena. Our study provides a model of how the VAB-1 Eph RTK modulates the growth cone structure to inhibit axonal outgrowth. We show that activated VAB-1 can inhibit an NCK-1/UNC-34 interaction by binding to the NCK-1 SH2 domain. We also show that NCK-1 and WSP-1 can physically interact and that VAB-1/NCK-1 and WSP-1 form a complex *in vitro*. We suggest that the VAB-1 Eph RTK can contribute to the termination of axon outgrowth by two methods: 1) The VAB-1/NCK-1/WSP-1 complex activates ARP-2/3 to change the actin growth cone dynamics to that of a branched structure thus reducing the number of filopodia, and 2) VAB-1 inhibits axon extension by inhibiting UNC-34/Ena's function in actin polymerization.

## Introduction

During development, axons navigate to their final destination by interpreting extracellular guidance cues through their growth cone. The Eph receptor tyrosine kinases (RTKs) and their ephrin ligands are involved in directing axons to their proper location [1], [2]. Studies in vertebrate systems have identified a number of effectors in the Eph RTKs signaling pathway in axon guidance [2]. However, the molecular mechanism of how Eph RTKs regulate axon guidance is still incomplete. This is partly due to the large number of Ephrins and Eph RTKs that can engage in crosstalk [2], [3]. The presence of a single Eph RTK, VAB-1, in *Caenorhabditis elegans* can simplify the analysis of the signal transduction events from the receptor. The *C. elegans* VAB-1 Eph RTK is required for various aspects of neuronal development, including neuroblast movements, and axon guidance [4], [5], [6], [7]. The molecules involved in VAB-1 signaling in axon guidance are still unknown. To resolve this issue, we used a genetic suppressor approach as well as a physical protein interaction approach and identified NCK-1, WSP-1/N-WASP, UNC-34/Ena, and the Arp2/3 complex as molecules regulated by VAB-1 signaling in axon guidance.

The Nck adaptor proteins are known actin cytoskeleton regulators, and have been shown to function downstream of several axon guidance receptors including Robo, Dcc and the Eph RTKs [8], [9], [10], [11]. Although the function of Nck has been studied in various organisms, the biological function of NCK-1 in *C. elegans* has only been recently explored [12]. Furthermore, what molecules interact with the *C. elegans* NCK-1 is still unknown.

The WASP protein family (WASP and N-WASP) are scaffolds that integrate multiple signaling pathways, leading to the formation of short branched actin filaments through the activation of the Arp2/3 complex [13]. The *C. elegans* N-WASP homolog, WSP-1, functions in neuronal cell migration and axon guidance [14], [15]. However, a connection between WSP-1 and a guidance receptor has not yet been established.

The Ena/VASP proteins are involved in actin-dependent movements including neuronal migration and axon guidance, and are known for their role in promoting filopodia formation [16]. In *C. elegans*, the Ena/VASP homolog UNC-34 is required for proper neuronal cell migration, axon guidance and filopodia formation [14], [17], [18], [19], [20]. Previous work has shown that Ena/VASP proteins are versatile in their developmental roles and function in both repulsive and attractive cues. For example Ena/VASP are effectors of receptors for repulsive cues such as SAX-3/Robo, UNC-5/Netrin receptor and EphB4, but they can as also act as effectors for attractive cues downstream of receptors such as UNC-40/DCC [21], [22], [23], [24], [25].

The Arp2/3 complex is a conserved family of actin nucleators and when activated results in the formation of an elaborate network of branched actin filaments similar to those found in lamellipodia [26], [27]. In *C. elegans*, the Arp2/3 complex is required for axon guidance, and the initiation of growth cone filopodia downstream of an unidentified axon guidance signal [15], [20].

In this paper, we describe some of the molecular events that allow the VAB-1 Eph RTK to regulate actin dynamics for axon guidance. We provide genetic and biochemical evidence to show that VAB-1 signals through NCK-1 and WSP-1/N-WASP, and negatively regulates UNC-34/Ena. We propose a model for PLM (Posterior lateral microtubule) axon termination whereby the VAB-1 Eph RTK is able to prevent axon extension by inhibiting growth cone filopodia formation. This is accomplished by negatively regulating the activity of the filopodia elongator UNC-34/Ena, and simultaneously activating Arp2/3 through a VAB-1/NCK-1/WSP-1 complex.

## Results

### VAB-1 signals through the *C. elegans* NCK-1 SH3/SH2 adaptor protein

**Figure 1:**
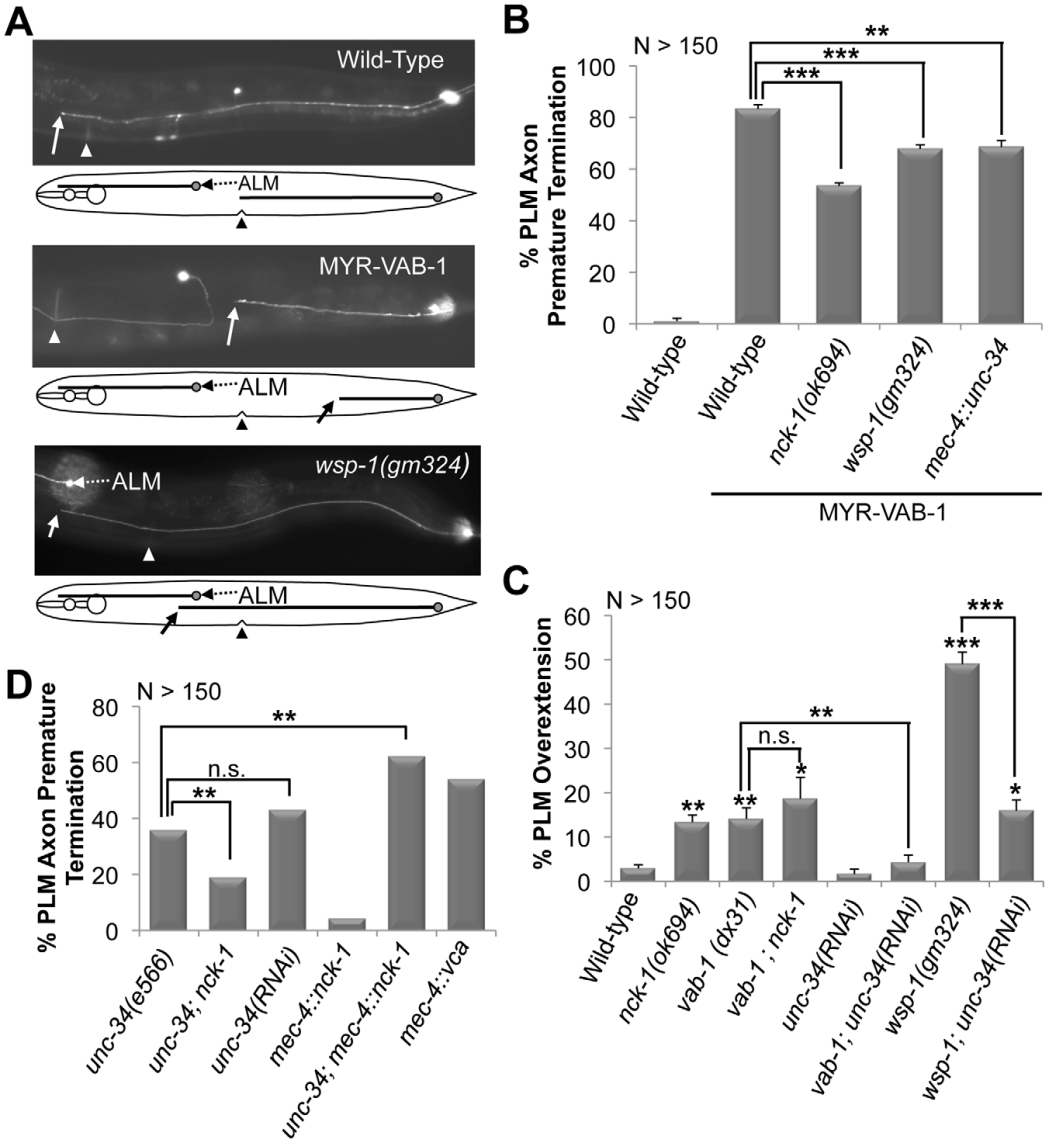
PLM defects in various backgrounds and their genetic interactions. (A) Young adults expressing *mec-4::gfp(zdIs5)*. Anterior is to the left. Solid arrow points to where the PLM axon ends. A line diagram that corresponds to the morphology of the neuron is shown below. In wild-type animals (top *gfp* panel) the PLM axons terminate at the middle (vulva region triangle). MYR-VAB-1 (middle *gfp* panel) causes PLM axons to terminate before reaching their target. *wsp-1(gm324)* animals (bottom *gfp* panel) have PLM axons that overshoot past the vulva (triangle) and ALM neuron (dashed arrow). (B) The *nck-1(ok694)* and *wsp-1(gm324)* alleles significantly reduced the early termination defects caused by MYR-VAB-1. Over expressing *unc*-34 in the PLMs also reduced the MYR-VAB-1 termination defect. (C) *vab-1*, *nck*-1 and *wsp-1* animals have PLM overextension defects. Reducing the levels of UNC-34 via tissue specific RNAi suppressed the PLM overextension defects of *vab-1(dx31)* and *wsp-1(gm324)*. (D) *unc-34(e566)* loss-of-function and tissue specific *unc-34* RNAi exhibit PLM axon termination. Over expression of NCK-1 in the mechanosensory neurons (*mec-4::nck-1*) caused low levels of the PLM early termination defects, but synergized in the *unc-34(e566)* background. Activating the Arp2/3 complex via the WSP-1 VCA domain (*mec-4::vca*) caused PLM axon termination defects. Error bars indicated the SEM, and significant differences between some of the strains were compared (using student's t-test), *P<0.05; **P<0.01; ***P<0.001; n.s. = not statistically significant. ‘N’ refers to the number of axons scored.

To identify VAB-1 Eph RTK effectors, we utilized transgenic animals carrying *mec-4::myr-vab-1 (quIs5)* which encodes a constitutively active VAB-1 tyrosine kinase (myristoylated-VAB-1 termed MYR-VAB-1) in the mechanosensory neurons [6]. In wild-type young adults, PLM neuron cell bodies are located in the tail region and have axons that stop at the centre of the animal (Figure 1A). We previously showed that *myr-vab-1* caused neuronal defects in the mechanosensory neurons, in particular the premature termination of PLM axons (Figure 1A, 1B) [6]. Since the MYR-VAB-1 behaves as a constitutively active VAB-1 RTK, we reasoned that mutations in effectors of the VAB-1 signal may suppress the neuronal defects. We used a candidate gene approach to examine genes with known roles in axon guidance and tested whether loss-of-function mutations could suppress the *myr-vab-1* PLM premature termination phenotype. We identified *nck-1* as a candidate effector of VAB-1 Eph RTK signaling. The *nck-1(ok694)* mutation partially suppressed the PLM axon premature termination (Figure 1B), indicating that other effectors are involved in the MYR-VAB-1 signaling. The *C. elegans* genome encodes for only one *nck-1* adaptor protein, and is most similar to the human Nck2 and *Drosophila* DOCK [12]. NCK-1 has all the domain features of the NCK adaptor proteins, including three SH3 domains followed by a single SH2 domain. We previously reported that the deletion allele *nck-1(ok694)* is predicted to be a null allele, thus all of our genetic work was carried out using the *ok694* allele [12].

If NCK-1 is an effector of VAB-1 signaling then we would expect the *nck-1* loss-of-function mutation to have a phenotype similar to that of the *vab-1* loss-of-function. Indeed, previous work showed that both *vab-1* and *nck-1* mutants have similar neuronal defects, including an overextension in PLM axons (Figure 1C) [6], [7], [12]. To further confirm that *nck-1* and *vab-1* are in the same pathway in the PLM neurons, we analyzed the effect of the double mutation on the PLM axons. The *vab-1; nck-1* double mutation did not enhance the PLM over extension phenotype (Figure 1C), indicating that NCK-1 and the VAB-1 Eph receptor function in the same pathway to guide the PLM axons.

**Figure 2:**
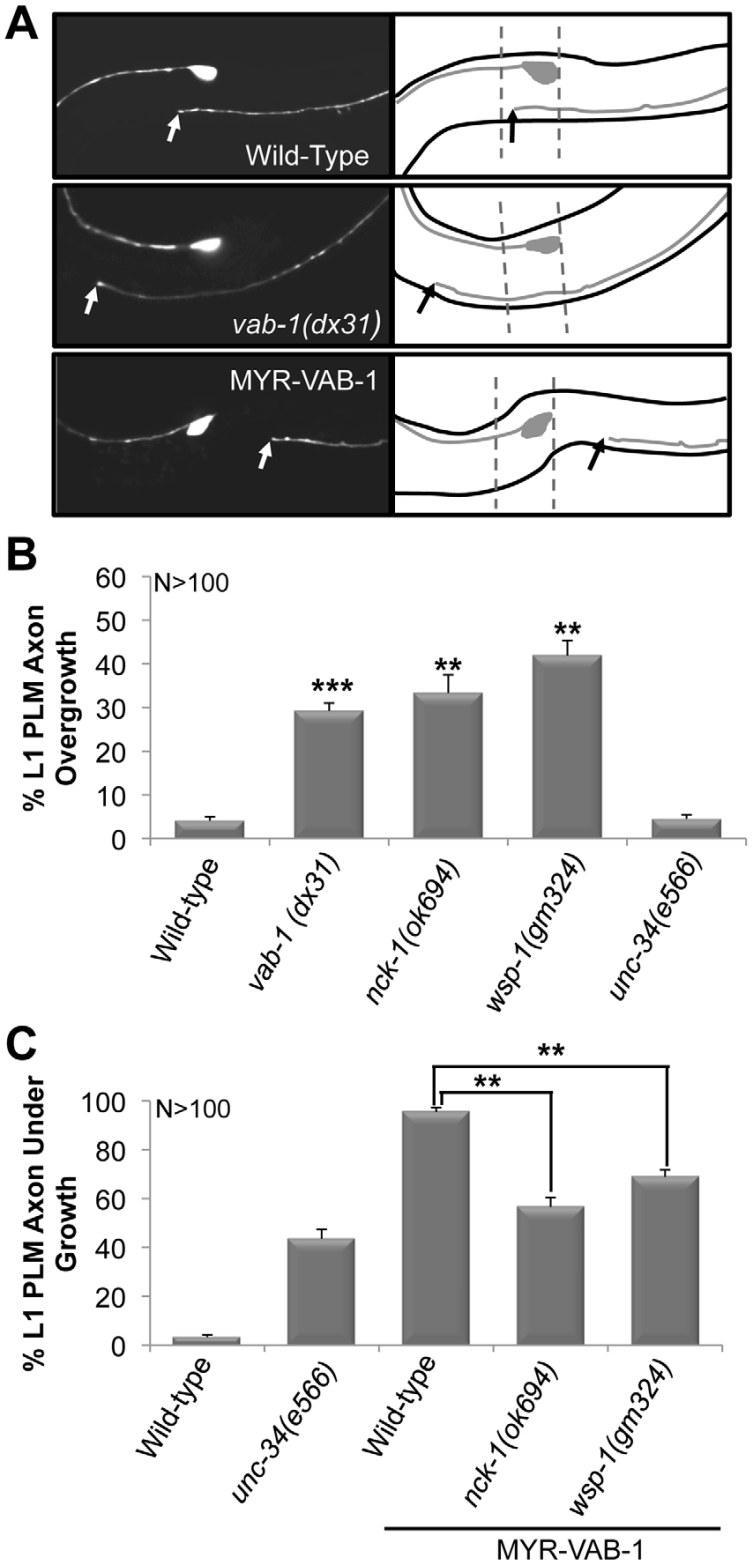
PLM over extension and early termination defects are observed at the L1 larval stage. (A) L1 larva expressing *mec-4;;gfp (zdIs5)*. Anterior is to the left. Solid arrow points to where the PLM axon ends. The ALM cell body and the anterior end of the PLM axon are shown in each panel. A line diagram corresponding to the morphology of the neuron is shown to the right. The dashed lines in all panels represent the wild-type PLM termination boundary. In wild-type L1s (top *gfp* panel) the PLM axons are between 103 and 114 µm in length, and terminate at a region anterior to the tip of the ALM cell body. *vab-1(dx31)* mutants (middle *gfp* panel) have L1 PLM axons that overgrow beyond the normal wild-type range. Animals that over express VAB-1 (MYR-VAB-1) (bottom *gfp* panel) have L1 PLM axons that are significantly shorter (undergrowth) than wild-type. (B) *vab-1*, *nck*-1 and *wsp-1* L1s have PLM axons that are overgrown beyond the normal range. (C) The *nck-1(ok694)* and *wsp-1(gm324)* alleles significantly reduced the undergrowth defects caused by MYR-VAB-1. Error bars indicated the SEM, and significant differences between some of the strains were compared (using student's t-test), **P<0.01; ***P<0.001. ‘N’ refers to the number of axons scored.

To determine if the PLM defects observed in *vab-1* and *nck-1* animals were present at an earlier stage, we examined the PLMs of the first larval stage (L1) (see Methods). Wild-type L1s had PLM axons that were 103–114 µm long, and terminated at a region anterior to the tip of the ALM cell body (93%) and is consistent with previous reports for L1 PLM lengths [28] (Figure 2A). Both *vab-1* and *nck-1* animals had PLM axons that significantly overgrew beyond the wild-type termination region (Figure 2A, 2B). This indicates that VAB-1 and NCK-1 are required at an early stage to prevent PLM axons from overgrowing beyond their normal termination region. We also showed that 96% of L1 *myr-vab-1* transgenic animals had PLM axons that were undergrown when compared to wild-type (Figure 2A, 2C). The PLM undergrowth defects caused by MYR-VAB-1 were significantly reduced by *nck-1(ok694)* (57%) (Figure 2C). These results are consistent with our analysis carried out in early adults, and further confirm that NCK-1 is an effector of VAB-1 signaling in PLM axon guidance.

### NCK-1 is expressed in the nervous system and co-localizes with VAB-1

**Figure 3:**
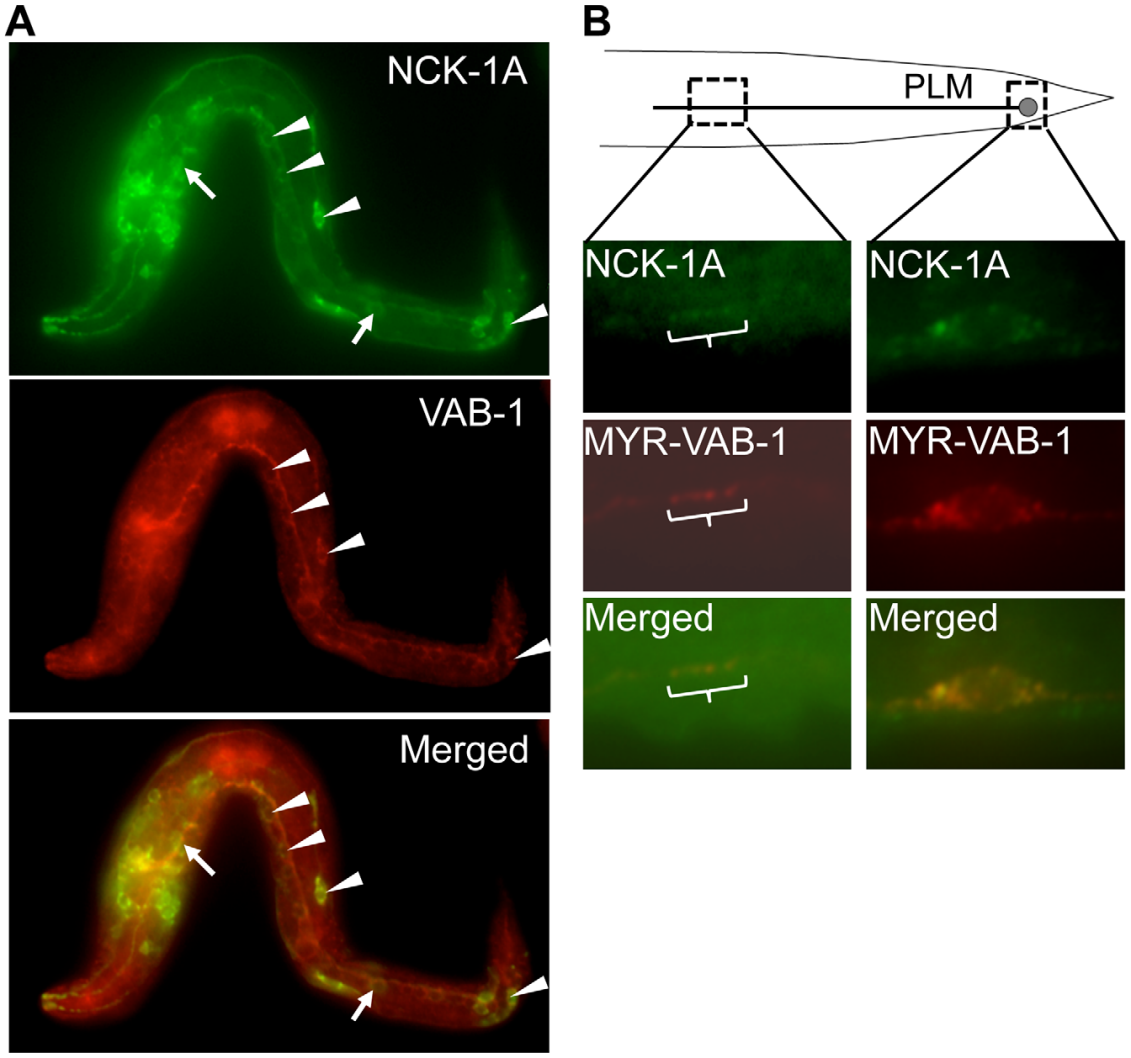
NCK-1 co-localizes with VAB-1. Anterior is to the left in all panels. (A–B) NCK-1 is in Green and VAB-1 is Red. (A) NCK-1 and VAB-1 co-localized in some cells. Arrow head points to cells where both NCK-1 and VAB-1 are co-localized, and the arrow points to cells that only express NCK-1. The translational *nck-1::gfp* transgene encodes the NCK-1A isoform and was detected using anti-GFP antibodies. Endogenous VAB-1 was detected using anti-VAB-1 antibodies (see experimental procedures). (B) NCK-1 and VAB-1 co-localized in the cell body and axon of the PLM neurons. *nck-1A::gfp* and *myr-vab-1* were expressed in the PLM under the *mec-4* promoter, and were detected using anti-GFP and anti-VAB-1 antibodies respectively.

We previously showed that NCK-1 is expressed in various tissues including the nervous system [12]. In addition, like VAB-1, NCK-1 can function cell autonomously in the mechanosensory neurons for PLM axon guidance [6], [12]. If NCK-1 and VAB-1 function in the same pathway during neuronal development, then they should be localized in the same cells. Indeed, NCK-1 and VAB-1 were co-localized in some of the neurons, consistent with the role of NCK-1 as an effector of VAB-1 (Figure 3A, 3B). However, the expression pattern of VAB-1 and NCK-1 did not overlap exactly, suggesting that both NCK-1 and VAB-1 have independent roles during development (Figure 3A). Expression of NCK-1-GFP and activated VAB-1 (MYR-VAB-1) in the mechanosensory neurons showed that NCK-1 did co-localize with activated VAB-1 in the PLM axon and cell body (Figure 3B).

### The NCK-1 SH2 domain interacts with VAB-1 phosphotyrosine Y673

**Figure 4:**
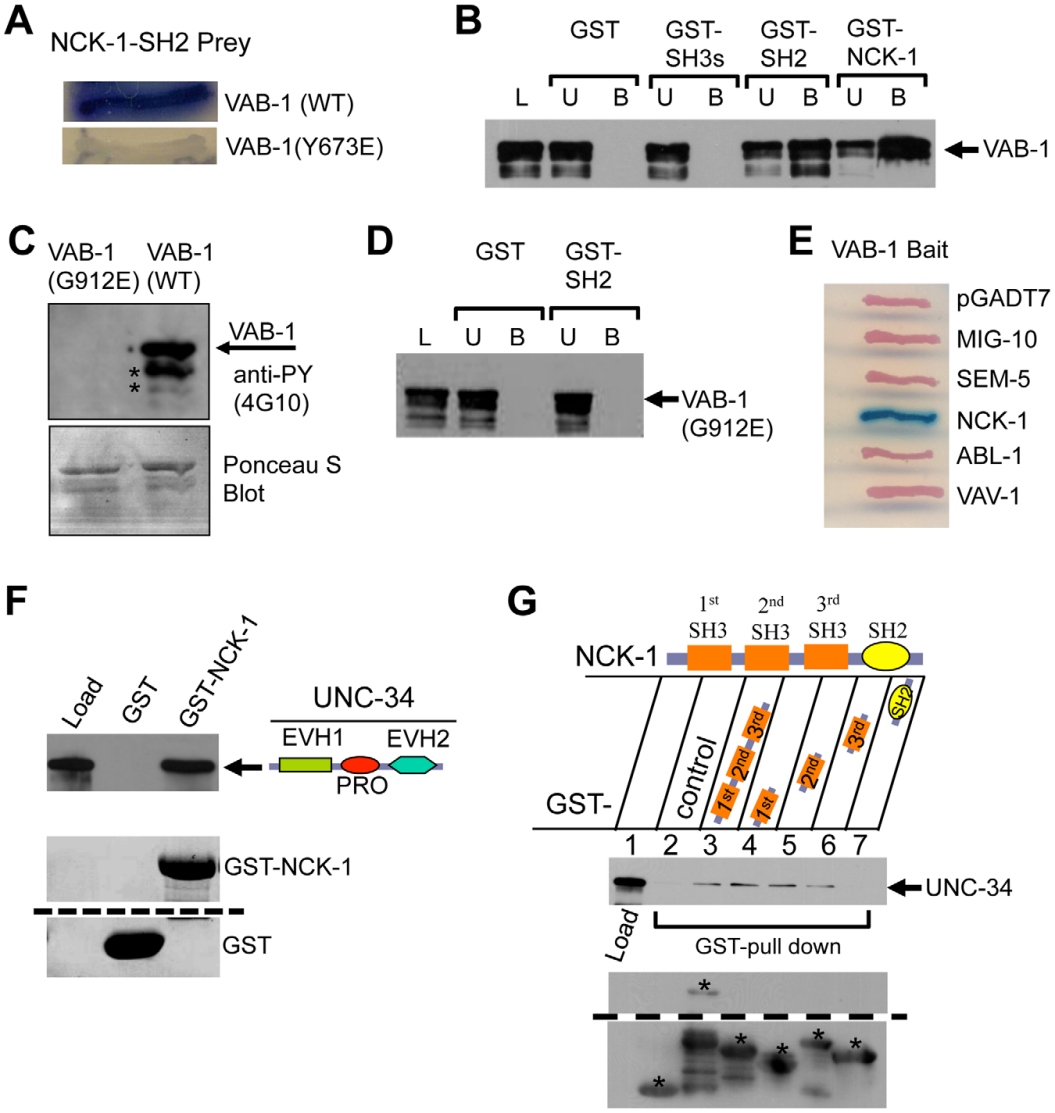
NCK-1 physically interacts with VAB-1 and UNC-34. (A) The NCK-1 SH2 domain binds to the Y673 of VAB-1. Yeast-two hybrid assays shows that the NCK-1 SH2 domain (Prey) can bind wild-type (WT) VAB-1 (669 aa–985 aa), but fails to interact when VAB-1 Y673 is changed to glutamic acid (Y673E). (B–C) GST pull-down assays. L = Load; U = unbound fraction; B = bound fraction. (B) Different NCK-1 domains fused to GST show that the SH2 domain is required for VAB-1 binding. The three SH3 domains alone do not bind VAB-1. (C) Bacterial expressed MBP-VAB-1 (intracellular region) has tyrosine kinase activity and shows autophosphorylation. The point mutation G912E in the VAB-1 kinase domain abolishes the VAB-1 kinase activity. The asterisks indicate break down products during purification. Phosphotyrosine was detected using anti-phosphotyrosine antibodies (4G10). The blot was stained with Ponceau S to show equal protein loading (below). (D) The binding of SH2 domain of NCK-1 to VAB-1 is kinase dependent. The kinase inactive VAB-1(G912E) failed to interact with the SH2 domain of NCK-1. (E) The NCK-1 SH2 domain shows high specificity for VAB-1. Yeast-two hybrid assays of other SH2 domains failed to interact with VAB-1. (F–G) GST-NCK-1 pull-down assays with MBP-UNC-34. (F) UNC-34 binds to NCK-1, both the proline rich (PRO) and EVH2 domains are required to bind NCK-1 (not shown). (G) Different GST-NCK-1 domains show that all SH3 domains can independently interact with full length UNC-34. The asterisks marks the correct protein fragment expected, and all other fragments below the marked are break down products. The dashed lines in E and F indicate a cropped region from the same blot.

In a parallel approach we used yeast two-hybrid screens to identify effectors of VAB-1/Eph RTK signaling and identified the full length NCK-1 as a binding partner of the VAB-1 intracellular kinase region. Yeast two-hybrid analysis showed that the NCK-1 SH2 domain is sufficient to bind VAB-1 and that VAB-1 tyrosine Y673 is crucial for the interaction with the NCK-1 SH2 domain (Figure 4A).

To further confirm the NCK-1/VAB-1 interaction we used GST-pull down assays. Deletion analyses confirmed that the SH2 domain is necessary and sufficient to bind VAB-1 (Figure 4B). Furthermore, the NCK-1 interaction required an active tyrosine VAB-1 kinase since the NCK-1 SH2 domain did not bind a kinase inactive version of VAB-1 (G912E) (Figure 4C, 4D). Since SH2 domains are known to bind phosphotyrosines we wanted to test how specific the NCK-1 SH2 domain is for VAB-1. We found that four other SH2 domains (MIG-10, SEM-5, ABL-1, VAV-1) were unable to bind VAB-1 (Figure 4E). In summary, NCK-1 interacts with VAB-1 in a kinase dependent manner, the interaction is mediated via the NCK-1 SH2 domain and the VAB-1 Y673 juxtamembrane tyrosine, and VAB-1 has high specificity for the NCK-1 SH2 domain.

### The Ena/VASP homolog UNC-34 can bind and inhibit NCK-1

How does VAB-1 cause the PLM to stop once the VAB-1 Eph RTK is activated and adaptor proteins such as NCK-1 bind the receptor? A previous report indicated that Ena/VASP was required for repulsion caused by EphB4 signaling in fibroblasts, but it was unclear how the signal was conveyed [24]. The Ena/VASP family are composed of an N-terminal EVH1 domain, a central PRO region and a C-terminal Ena/VASP homology II domain (EVH2) [16]. We asked if NCK-1 could be the link between the Eph RTK and Ena/VASP. We first tested if NCK-1 and UNC-34 can directly interact. *In vitro* binding assays with bacterially expressed NCK-1 and UNC-34 confirmed that both proteins do indeed physically interact (Figure 4F, 4G). Furthermore, we found that the PRO-EVH2 domains are required together to bind NCK-1 (data not shown). We also showed that all three NCK-1 SH3 domains were able to bind UNC-34 (Figure 4G).

While *nck-1* and *vab-1* animals have overextended PLM axons, *unc-34* animals have the opposite phenotype and have PLM axons that terminate prematurely (Figure 1D, Figure 2C). This suggests that UNC-34 is involved in PLM axon extension, and reflects a known role of Ena/VASP in actin filament formation and elongation [16], [29]. To understand the genetic nature of the interaction between *nck-1* and *unc-34*, we analyzed the *nck-1(ok694); unc-34(e566)* double and found that *nck-1* partially suppressed the *unc-34* PLM termination defect, while *unc-34* did not suppress the *nck-1* overgrowth (Figure 1D, and data not shown). This suggests that, in PLM axon outgrowth, *unc-34* may negatively regulate *nck-1*. To provide further evidence for this genetic interaction we over expressed NCK-1 (*mec-4::nck-1*) in the PLM neurons of *unc-34(e566)* animals and this resulted in a synergistic enhancement of the *unc-34* PLM termination phenotype (Figure 1D). Although we cannot conclusively rule out that *nck-1* inhibits *unc-34*, overall, our results suggest that UNC-34 can inhibit the function of NCK-1 and may do so by physically binding to it.

### VAB-1 disrupts the NCK-1/UNC-34 interaction and negatively regulates UNC-34

**Figure 5:**
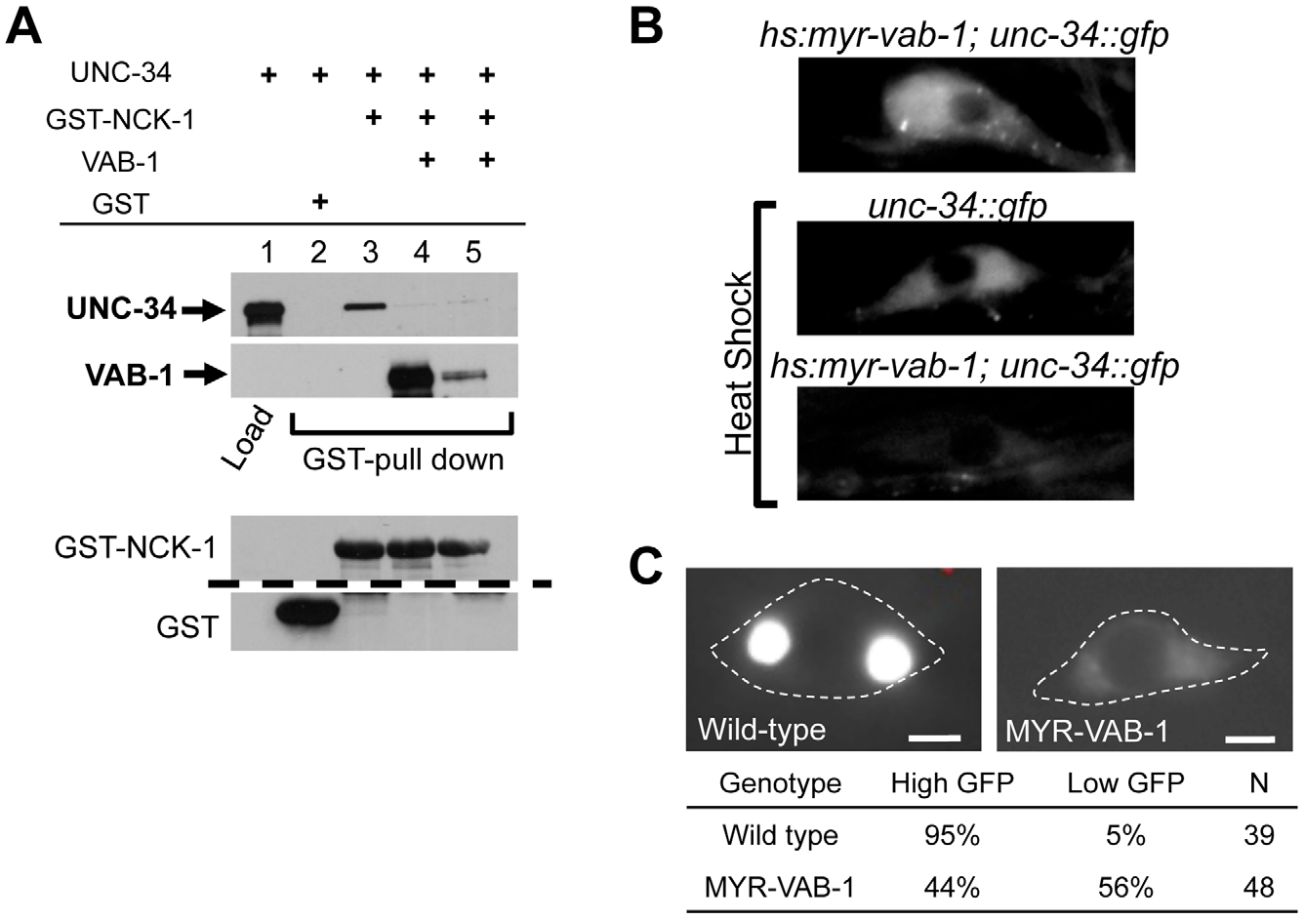
VAB-1 inhibits the UNC-34/NCK-1 complex and negatively regulates UNC-34 protein levels. (A) NCK-1 (GST) pull-down experiments. NCK-1 can pull-down UNC-34 (lane 3). Adding VAB-1 (either co-expressed (high VAB-1), lane 4, or mixing (low VAB-1), lane 5) inhibits the interaction between NCK-1 and UNC-34. VAB-1, NCK-1 and GST protein levels shown below, the dashed line indicates a cropped region from the blot. Tagged protein fusions used: GST-NCK-1, HIS-VAB-1, and MBP-UNC-34. Proteins were detected with antibodies to GST, MBP or VAB-1. (B) Inducing hyperactive MYR-VAB-1 (*quIs16*) via a heat shock promoter reduces the levels of UNC-34::GFP. *unc-34::gfp* transgenic animals had a GFP relative mean intensity of 1±0.07 (N = 12) under heat shock conditions, while *hs:myr-vab-1;unc-34::gfp* animals had a GFP relative mean intensity of 0.56±04 (p<0.01; student's t-test; N = 16) under the same conditions. All panels show UNC-34::GFP image of the CAN neuron. ‘N’ refers to the number of animals. (C) Images show PLM cells expressing *mec-4::unc-34::gfp*. In wild-type animals (left panel), the PLM shows high levels of UNC-34 expression and in contrast, the UNC-34 intensity is reduced in *mry-vab-1* transgenic animals (right panel). Images were taken at identical exposure settings; dashed line outlines the PLM cell body; ‘N’ = number of animals; scale bar equals 2 µm.

Since UNC-34 and NCK-1 physically interact, we wanted to examine whether VAB-1, NCK-1 and UNC-34 could form a complex *in vitro*. Surprisingly, although UNC-34 can bind strongly to NCK-1, the introduction of VAB-1 abolished the binding between UNC-34 and NCK-1 (Figure 5A Lane 4, 5). This result suggests that VAB-1 might be inducing its effect at the growth cone membrane by relieving the inhibition of NCK-1 that is caused by UNC-34. To provide *in vivo* support of this we over expressed UNC-34 in the mechanosensory neurons (*mec-4::unc-34*) and it significantly reduced the MYR-VAB-1 PLM premature termination phenotype (Figure 1B).

To gain more insight into the interaction between VAB-1 and UNC-34, we sought to analyze the effect of the *vab-1;unc-34* double on PLM axons. We found that the *vab-1;unc*-34 double mutant is synthetic lethal (data not shown), so we used a mechanosensory specific *unc-34* RNAi approach (see experimental procedures). The *unc-34(RNAi)* strain had PLM termination defects that were similar to *unc-34(e566)* (Figure 1D). Analysis of the *vab-1;unc-34(RNAi)* double showed that reducing the levels of *unc-34* can rescue the PLM overextension defects seen in *vab-1(dx31)* (Figure 1C), which is consistent with *vab-1* inhibiting *unc-34* function. Since the genetic data suggested that *vab-1* negatively regulates *unc-34*, we questioned if the activation of VAB-1 could affect the expression and/or localization of UNC-34. Induction of MYR-VAB-1 via heat shock promoter did not change the localization of UNC-34, but instead resulted in the reduction of UNC-34::GFP levels compared to wild-type animals (Figure 5B). To test whether VAB-1's negative regulation can function cell autonomously in the PLMs, we expressed UNC-34::GFP only in the mechanosensory neurons (via *mec-4* promoter) and it is expressed at high levels. When we introduce constitutively active VAB-1 only in the touch neurons (*mec-4::myr-vab-1*) it reduced the UNC-34::GFP levels significantly (Figure 5C).

In summary, our binding assays and genetic analyses show that VAB-1 activation results in binding NCK-1 which in turn blocks the UNC-34 binding to NCK-1, freeing NCK-1 from the negative influence of UNC-34 and in addition VAB-1 negatively regulates UNC-34 protein levels.

### WSP-1 is an effector for VAB-1/NCK-1

Since mammalian Nck is known to physically bind and activate N-WASP to regulate actin filaments through the Arp2/3 complex [8], [30], [31], we questioned whether VAB-1 is linked to the cytoskeleton through WSP-1/N-WASP. If WSP-1 acts downstream of VAB-1, then the *wsp-1* mutants should suppress the PLM termination defect caused by MYR-VAB-1. Two *wsp-1* alleles are predicted to affect the WSP-1 protein. The *wsp-1(tm2299)* is not well characterized, but is homozygous lethal and is predicted to be a null allele. The embryonic lethality is due to *wsp-1* pleiotropy as WSP-1 is also required for cytokinesis during embryogenesis [14]. The *wsp-1(gm324)* allele is a well characterized deletion that removes exons 2 and 3, furthermore, no WSP-1 protein nor mRNA can be detected, therefore *wsp-1(gm324)* is a strong loss-of-function allele [14]. *wsp-1(gm324)* displays some embryonic and larval lethality but can be maintained as a homozygote [14], [15], [32]. We chose to use the *wsp-1(gm324)* allele as it allowed us to bypass the embryonic lethality associated with the *wsp-1* null allele. We found that *wsp-1(gm324)* could significantly suppress the MYR-VAB-1 PLM termination defect in young adults and L1s (Figure 1B, Figure 2C).

If WSP-1 is an effector of VAB-1 signaling then we would expect to see neuronal defects similar to *vab-1* animals. It was previously reported that the *wsp-1(gm324)* had weak axon guidance defects, such as in the PDE and VD/DD neurons [15]. We report here that approximately 50% of *wsp-1(gm324)* animals have overextended PLM defects in young adults, and 42% PLM axon overgrowth in L1s (Figure 1C, Figure 2B). Since the *wsp-1* PLM overextension frequency is much greater than *vab-1* (Figure 1C), it implies that WSP-1 also functions independent of VAB-1 for PLM axon guidance. We also found that the *vab-1(dx31);wsp-1(gm324)* double mutants are synthetic lethal (data not shown), which is consistent with WSP-1 functioning in parallel pathways with VAB-1.

The presence of WSP-1 in the VAB-1 signaling pathway suggests the possibility that the PLM termination phenotype caused by MYR-VAB-1 could be due to the activation of the Arp2/3 complex. WSP-1, like its mammalian counterpart, is composed of an N-terminal Ena/VASP homology I domain (EVH1; also known as WASP-homology-1 domain (WH1)), a central section containing a basic region (BR), a GTPase binding domain (GBD) and a proline-rich region (PRO), and a C-terminal with two verprolin homology domains (V; also known as WH2), a cofilin homology domain (C) and an acidic domain (A) [13], [14], [32] collectively known as the VCA region. The C-terminal VCA regions of both WSP-1 and N-WASP have been shown to be sufficient for activating the Arp2/3 complex *in vitro* [32], [33]. We utilized the C-terminal VCA region of WSP-1 to selectively activate the Arp2/3 complex in the mechanosensory neurons (*mec-4::wsp-1^vca^*). The WSP-1^VCA^ caused PLM premature termination defects that were very similar to MYR-VAB-1 (Figure 1D).

The activation of high levels of the Arp2/3 complex produces extensive short branched actin networks that prevent the formation of filopodia, and hence can inhibit axon extension [34], [35]. Ena/VASP, on the other hand, promotes axon extension through filopodia formation and elongation [16], [17], [20], [29]. Thus, activation of Arp2/3 complex and UNC-34/Ena have opposite roles in the axon growth cone, and perhaps Arp2/3 complex activation can antagonize the function of UNC-34/Ena. Since WSP-1/N-WASP is an activator of the Arp2/3 complex, we wanted to test genetically if *wsp-1* can antagonize *unc-34* function. Due to the synthetic lethality of *wsp-1; unc-34* double mutants [14], [36], we analyzed the PLM axons in *wsp-1; unc-34(RNAi)* animals. Tissue specific *unc-34* RNAi resulted in the partial suppression of PLM overextension defects caused by *wsp-1(gm324)* (Figure 1C), consistent with WSP-1/Arp2/3 activity antagonizing UNC-34 function.

In summary, we show that WSP-1 functions in PLM axon termination, through various signaling pathways, including the VAB-1 Eph RTK. Our results suggest that MYR-VAB-1 is exerting its effect by activating the Arp2/3 complex through WSP-1. We also suggest that WSP-1 can antagonize UNC-34 function by activating the Arp2/3 complex.

### VAB-1 enables WSP-1 to outcompete UNC-34 for NCK-1 binding

**Figure 6:**
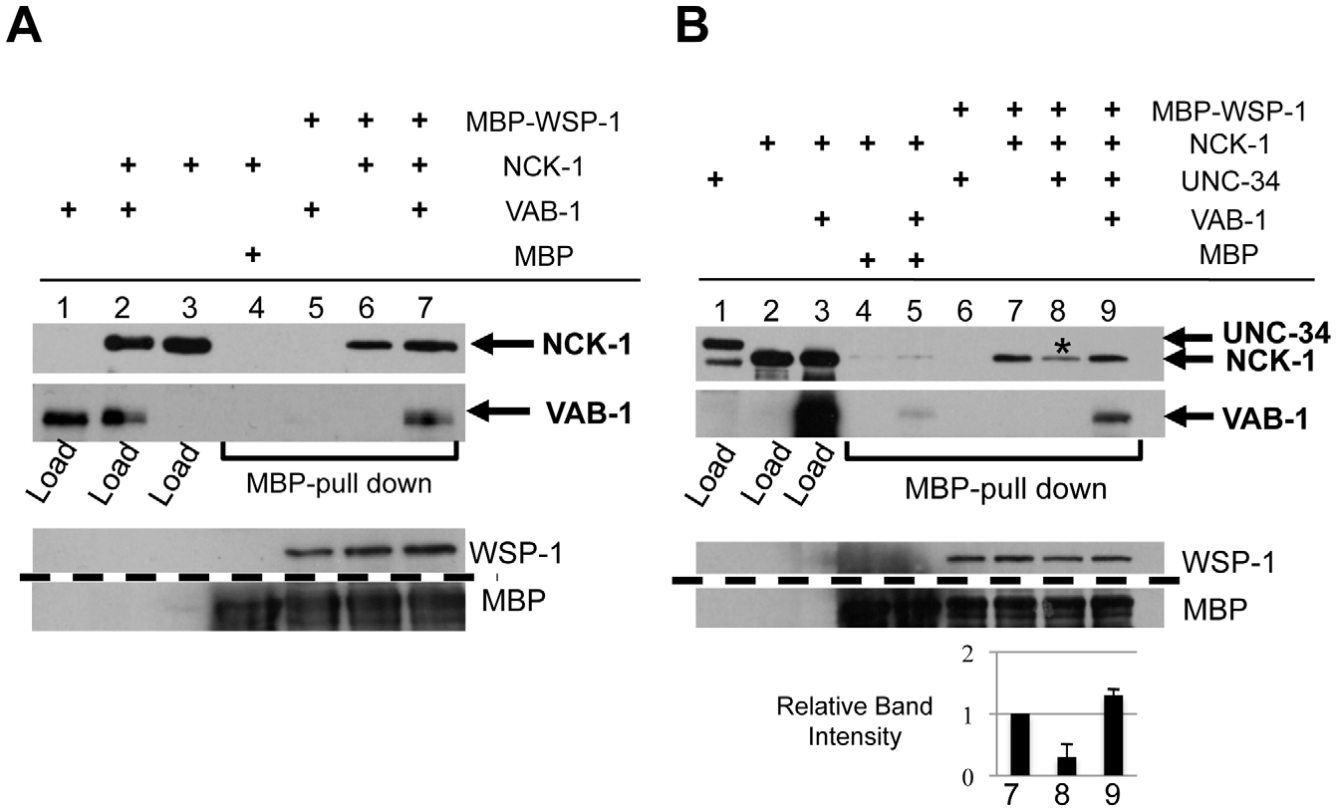
VAB-1, NCK-1, and WSP-1 interact in a complex. (A–B) WSP-1 (MBP) pull-down experiments. (A) VAB-1/NCK-1/WSP-1 can form a complex. WSP-1 does not pull-down VAB-1 (lane 5), WSP-1 can pull down NCK-1 (lane 6), and WSP-1 can pull down VAB-1 only in the presence of NCK-1 (compare lane 5 and lane 7), (B) VAB-1 enables WSP-1 to outcompete UNC-34 for NCK-1 binding. WSP-1 does not pull down UNC-34 (lane 6). WSP- 1 pulls down NCK-1 (lane 7, NCK-1 relative intensity 1.0) and adding UNC-34 reduces the level of interaction between NCK-1 and WSP-1 (lane 8 (asterisks), NCK-1 relative intensity 0.3±0.2). UNC-34 is not detected in lane 8 suggesting that NCK-1/UNC-34/WSP-1 do not form a complex. Adding VAB-1 prevents UNC-34 from inhibiting the interaction between NCK-1 and WSP-1 (lane 9, NCK-1 relative intensity 1.3±0.1). Protein levels shown below, dashed line indicates a cropped region from the blot. Tagged protein fusions used: MBP-WSP-1, GST-NCK-1, HIS-VAB-1 and GST-UNC-34. Proteins were detected with antibodies to GST, MBP or VAB-1.

We used *in vitro* binding assays to ask whether VAB-1, NCK-1 and WSP-1 could form a complex. WSP-1 was able to bind NCK-1 (Figure 6A, Lane 6), but not VAB-1 (Figure 6A, Lane 5). However, WSP-1 was able to pull down VAB-1 in the presence of NCK-1, indicating that a VAB-1/NCK-1/WSP-1 complex can occur (Figure 6A, Lane 7).

Since NCK-1 is able to bind both UNC-34 and WSP-1, we wanted to determine whether all three molecules can form a complex, or do UNC-34 and WSP-1 compete for NCK-1 binding. We first confirmed that WSP-1 was unable to bind UNC-34 (Figure 6B, Lane 6). We found that although WSP-1 binds NCK-1, the presence of UNC-34 resulted in a 70% reduction of the NCK-1/WSP-1 complex (Figure 6B, Lane 8). This shows that UNC-34 can effectively compete with WSP-1 for NCK-1 binding. Furthermore we could not detect NCK-1/UNC-34/WSP-1 in a complex (Figure 6B, Lane 8). Interestingly, adding VAB-1 to the binding interaction increased the level of NCK-1 binding to WSP-1, indicating that VAB-1 eliminated UNC-34's ability to compete for NCK-1 binding (Figure 6B, Lane 9). In summary, our binding assays show that VAB-1, NCK-1 and WSP-1 form a complex, that UNC-34 competes with WSP-1 for NCK-1 binding, and that VAB-1 enables WSP-1 to outcompete UNC-34 for binding to NCK-1.

### VAB-1 signaling inhibits filopodia on the PLM growth cone

**Figure 7:**
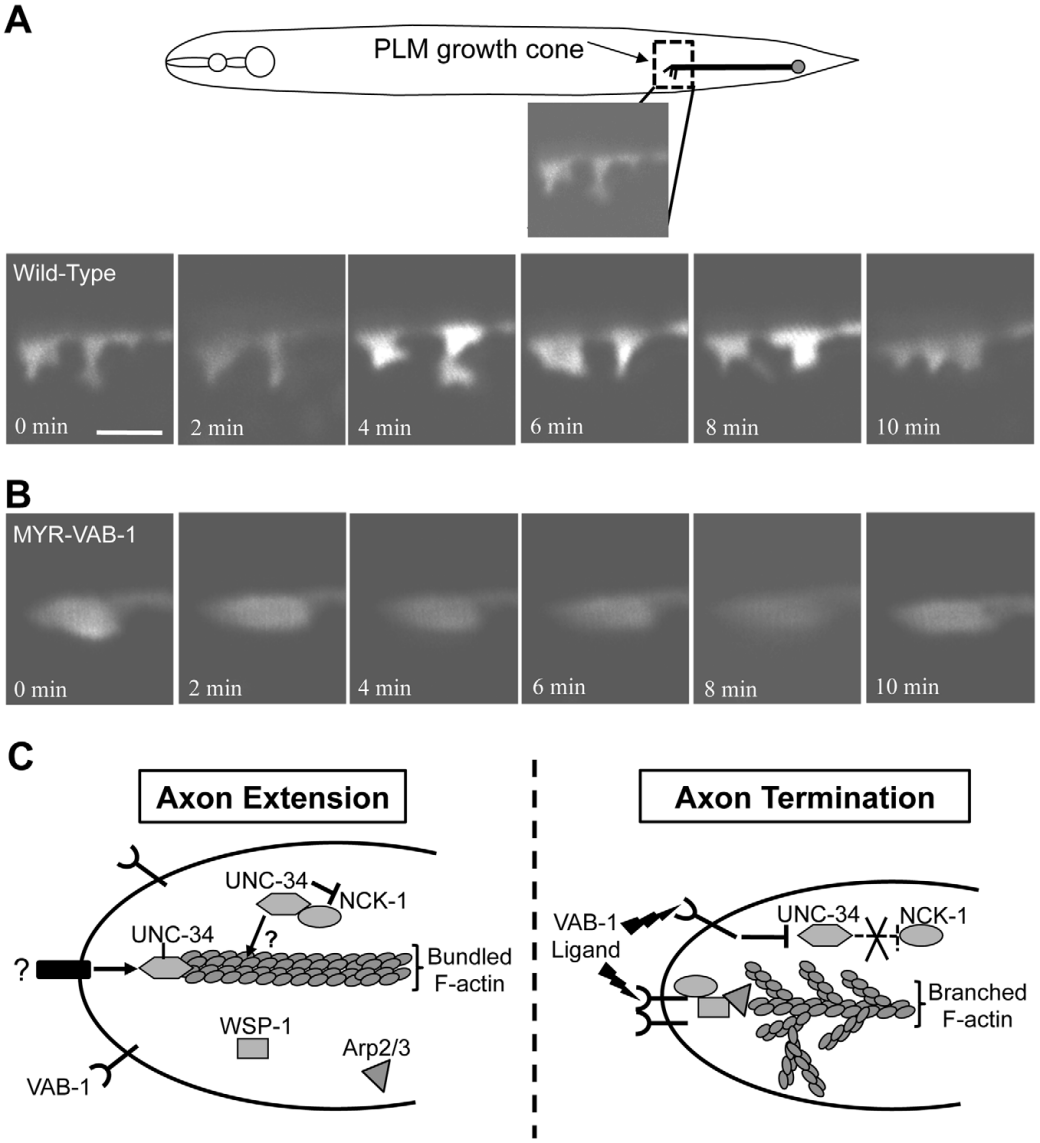
VAB-1 activation affects PLM growth cone dynamics. (A–B) Series of time-lapse images of newly hatched L1 PLM growth cones as they migrate anteriorly. Scale bar represents 2 µm. (A) Wild-type PLM growth cones exhibit dynamic changes and display multiple filopodia protrusions. (B) Transgenic *myr-vab-1* animals have PLM growth cones that are less dynamic and mostly void of any protrusions. (C) A model of how VAB-1 induces its termination effect during PLM axon guidance. (Left) In the absence of VAB-1 activation, UNC-34/Ena promotes axon extension through the polymerization of actin filaments and forming filopodia. UNC-34/Ena also physically binds to and inhibits NCK-1's role with WSP-1. It is possible that the UNC-34/NCK-1 heterodimer could work together in promoting actin polymerization downstream of other receptors and is indicated by question marks (?). (Right) Genetic and molecular data suggest that VAB-1 inhibits UNC-34 function. Activation of VAB-1 contributes to stopping axon outgrowth by binding to the NCK-1 SH2 domains, which disrupts the interaction between NCK-1 and UNC-34. VAB-1 over expression can reduce UNC-34 protein levels thereby preventing further actin filament polymerization. Furthermore, VAB-1, NCK-1 and WSP-1 can now form a complex and induce high levels of Arp2/3 activation to form an extensive network of short, branched actin filaments. The combination of inhibiting actin filament polymerization and increasing short, branched networks stop the formation of new filopodia. The net result is the termination of axon extension in response to VAB-1 signaling.

The VAB-1 RTK effectors NCK-1 and WSP-1 are known actin regulators and therefore implicate VAB-1 signaling in regulating actin cytoskeleton for axon guidance. To confirm this, we monitored the PLM growth cone of wild-type and *myr-vab-1* transgenic animals at the time of hatching. In wild-type animals, most of the PLM growth cones exhibited dynamic changes and had many filopodia protrusions (70%; N = 20 movies) (Figure 7A, Video S1). Transgenic *myr-vab-1* animals, on the other hand, had growth cones that were less dynamic and were usually void of filopodia like structures with only 25% (N = 16 movies) showing some filopodia structures (Figure 7B, Video S2). Since our molecular and genetic data suggest that VAB-1 inhibits UNC-34/Ena function we also observed the growth cones of *unc-34(e566)* animals. We found that *unc-34(e566)* mutants, like *myr-vab-1* animals, had growth cones void of filopodia structures with only 25% displaying filopodia structures (N = 12 movies; not shown). Our results show that activated VAB-1 can affect the PLM growth cone morphology by inhibiting filopodia formation.

## Discussion

We previously reported a functional role for VAB-1 as a receptor for a repellent or stop signal in PLM axon guidance [6]. Here we describe some of the molecular events involved in VAB-1 signaling that allow the regulation of actin dynamics for PLM axon guidance. Our genetic and *in vitro* interaction analysis identified NCK-1, WSP-1 and UNC-34 as molecules regulated by VAB-1 Eph RTK signaling. Our data supports a model in which VAB-1 suppresses axon extension by negatively regulating UNC-34, and activating the Arp2/3 complex through a VAB-1/NCK-1/WSP-1 complex. Furthermore, using time-lapse analysis we show that activation of VAB-1 inhibits filopodia formation in the PLM growth cone.

### NCK-1 and Eph RTK signaling

Our results show that the *C. elegans* NCK-1 adaptor protein is an effector of the VAB-1 RTK signal *in vivo*. Several lines of evidence indicate that VAB-1 and NCK-1 act together to regulate axon guidance. First, *nck-1* and *vab-1* animals have similar neuronal defects. Second, NCK-1 and VAB-1 physically interact and co-localize in similar neuronal cells and axons. Finally, the *nck-1* loss-of-function suppresses the defects caused by the constitutively active VAB-1. We found that NCK-1 binds the VAB-1 juxtamembrane tyrosine Y673 (YEDP) via its SH2 domain in a VAB-1 kinase dependent manner. This is consistent with the published binding specificity of the Nck SH2 domain, as well as reports of Nck1 binding to the second juxtamembrane tyrosine residue (YEDP) in EphA3 (Y602) and EphA2 (Y594) [37], [38], [39]. Interestingly, Nck adaptors have been reported to function downstream of Eph RTKs but it appears that the activated EphA RTKs are direct targets of Nck adaptors [38], [39], [40], [41], whereas Nck may indirectly interact with EphBs [11], [42], [43]. Considering that the intracellular region of VAB-1 is more similar to EphA receptors [4], our results in *C. elegans* provides relevant insight into how mammalian EphA receptors could regulate the actin cytoskeleton for axon guidance.

### Ena/VASP in Eph RTK signaling

The Ena/VASP protein family is required in processes that involve dynamic actin remodeling such as platelet shape change, axon guidance and Jurkat T cell polarization [44]. The ability of Ena/VASP proteins to remodel actin stems from their ability to polymerize actin, which is required for filopodia formation and elongation [16], [45], [46]. In *C. elegans*, UNC-34/Ena functions in neuronal cell migration, axon guidance and epithelial filopodia formation [14], [18], [19], [20]. Our results further confirm the role of UNC-34 in axon extension, because we show that the *unc-34(e566)* PLM axons terminated prematurely. The cause of early termination is likely due to a reduction of filopodia elongation in the growth cone, resulting in the persistence of more densely branched filaments that can slow axon migration. This is supported by the finding that *unc-34* mutants have fewer filopodia structures on growth cones, and a reduced rate of growth cone migration [20] (this work and our unpublished observations). In addition, mammalian studies show that depletion of Ena/VASP generates shorter and more densely branched filaments [47].

We propose that VAB-1 negatively regulates UNC-34 for PLM termination. This is supported by our observations that: 1) the loss-of-function *unc-34* resulted in PLM axon defects similar to the hyperactive MYR-VAB-1; 2) over expressing UNC-34 in the PLM partially suppressed the MYR-VAB-1 phenotype; 3) tissue specific *unc-34* RNAi suppressed the *vab-1* PLM overextension defects; and 4) over expressing VAB-1 reduced the UNC-34 protein levels. Although we do not know the mechanism of the reduction of the UNC-34 protein levels displayed in the hyperactive VAB-1, it is possible that UNC-34, when removed from its adaptor NCK-1, is more prone to degradation. In this case NCK-1 may play a dual role and may also promote UNC-34 function as well. It is also likely VAB-1 signaling could affect the *unc-34* transcriptional level. Future experiments should resolve how VAB-1 regulates UNC-34 protein levels.

Our finding that VAB-1 negatively regulates UNC-34/Ena is different from a previous report that shows mammalian EphB4 as an activator of Ena/VASP [24]. In fibroblast cells, the EphB4 receptor is thought to activate Ena/VASP to destabilize lamellipodia during cell repulsion and likely does so by promoting elongated actin filaments rather than a branched actin filament network. Although the Eph receptor signal transduction to Ena or UNC-34 is opposite (activates vs. inhibits) the role for UNC-34/Ena is conserved, because in both cases UNC-34 or Ena/VASP promotes actin filament elongation.

### A VAB-1/NCK-1/WSP-1 complex regulates the actin cytoskeleton

Our results provide evidence that VAB-1/Eph RTK can regulate the actin cytoskeleton through its interaction with NCK-1 and WSP-1. This is based on our observation that *vab-1*, *nck-1* and *wsp-1* mutants share the same phenotype of PLM axon overextension, that both *nck-1* and *wsp-1* were able to partially suppress the MYR-VAB-1 PLM termination defect, that VAB-1, NCK-1 and WSP-1 are able to form a complex *in vitro*, and that the activation of the Arp2/3 complex via the WSP-1 VCA domain resulted in PLM termination defects similar to MYR-VAB-1. The role of N-WASP as a negative regulator of axon elongation has been shown by two separate reports, where the reduction of N-WASP resulted in the enhancement of axon elongation [48], [49]. This phenotype is reminiscent of the PLM overextension defects we observed in *wsp-1* animals. There have been conflicting reports on the role of the Arp2/3 complex in axon elongation, where some reports suggest that the Arp2/3 complex acts as a negative regulator of axon elongation [34], [49], while other reports show that the Arp2/3 complex is required for axon elongation [20], [50]. A paper by Ideses et al. (2008) provided a potential resolution to this paradox by looking at the characteristics of actin assembly in the presence of variable amounts of Arp2/3 complex *in vitro* [35]. It is proposed that high levels of the Arp2/3 complex prevent the formation of filopodia bundles by promoting the extensive branching networks of actin with short tips. On the other hand, at low concentrations of Arp2/3 the actin filaments have longer tips and are further apart making it easier to form filopodia bundles [35]. Therefore, it would be expected that the complete elimination of Arp2/3 would prevent any neurite elongation. Similarly, the excessive activation of Arp2/3 would also prevent neurite elongation due to the increased levels of short, branched networks of actin filaments. In the *C. elegans* epithelial cells *unc-34* and *wsp-1* function redundantly for epithelial cell migrations [14]. However our results in PLM neurons suggest that WSP-1 and UNC-34 have opposite roles. Why the apparent paradox? This is reminiscent of what has been observed for Ena/VASP proteins where some reports suggest Ena/VASP promotes actin dependent processes while others suggest Ena/VASP may inhibit actin dependent processes [51]. While the growth cones on axons and the leading edge of epithelial cells both require actin for movement, they might not be identical in the way the cell moves forward. Proteins such as Ena/VASP, N-WASP, and Arp2/3 are thought to promote actin polymerization, however these proteins also change the geometry of the actin filament network in addition to promoting actin assembly. Therefore the overall effects of such changes in the actin network may not be easy to predict with respect to cell movement since various concentrations of these actin regulators could lead to activation or inhibition of filopodia. Since WSP-1/N-WASP is an activator of the Arp2/3 complex and different levels of Arp2/3 can elicit different behaviors, WSP-1 may also have opposite effects depending on its level of activity. In addition, while most of our results are based on the PLM neurons it is very likely the roles of UNC-34 and WSP-1 and how they are regulated will be different in other neurons.

N-WASP has been shown to interact in a complex with the mammalian EphB2, through the adaptor molecule intersectin [52]. Furthermore, this complex of EphB2, intersectin and N-WASP is required for dendritic spine formation, which consists mainly of a meshwork of branched filaments caused by the activation of the Arp2/3 complex [52]. *C. elegans* intersectin (ITSN-1) is expressed in the nervous system, and it is enriched in presynaptic regions and has roles in neurotransmission [53]. Future work will determine whether the VAB-1/Eph interacts with ITSN-1 to connect WSP-1. Our current work shows that the VAB-1 Eph RTK can signal through WSP-1/N-WASP through a different adaptor molecule, NCK-1, and we propose, like the mammalian intersectin adaptor, this complex activates Arp2/3 to promote branched actin.

### Model for VAB-1 signaling in the PLM to stop axon growth

We propose a model of how the proteins VAB-1, NCK-1, UNC-34, WSP-1 and Arp2/3 function in axon growth cones for extension and termination (Figure 7C). During PLM axon outgrowth, the growth cone is stimulated by an attractive cue that results in the accumulation of UNC-34/Ena at the growth cone. The result is a net forward movement due to the role of UNC-34/Ena in inhibiting actin capping proteins, and allowing filopodia elongation by polymerizing F-actin at the leading edge. In addition, UNC-34/Ena binds to the NCK-1 SH3 domains to prevent it from interacting with WSP-1 and participating in a signaling pathway(s) that would otherwise inhibit axon extension. It is also possible that the UNC-34/NCK-1 heterodimer could function together for actin polymerization or that NCK-1 binding could stabilize the UNC-34 protein. In this case NCK-1 acts positively with UNC-34. However, since *unc-34* and *nck-1* mutants have opposite PLM axon phenotypes, it suggests that *nck-1*'s role in axon outgrowth is more dispensable or redundant than its role in axon termination. Once the VAB-1/Eph RTK receives the signal to inhibit axon extension, VAB-1 is autophosphorylated and provides a docking site (Y673) for NCK-1. The NCK-1-SH2 domain binds the activated VAB-1 receptor and this disrupts the interaction between NCK-1 and UNC-34 to release the inhibitory effect of UNC-34 on NCK-1. Through an unknown mechanism, we also show that VAB-1 negatively regulates the UNC-34/Ena protein levels. VAB-1/NCK-1 can now recruit and activate WSP-1 and all three proteins form a complex, which results in high levels of Arp2/3 activation, ultimately leading to a branched meshwork of actin filaments. The combined actions of VAB-1/Eph blocking UNC-34/Ena activity, while activating Arp2/3 through NCK-1/WSP-1 contributes to the molecular events required to stop the growth cone forward movement.

## Materials and Methods

### Strains

All *C. elegans* strains were manipulated as described by Brenner [54]. All alleles were isolated in the standard wild type Bristol strain N2. All experiments were performed at 20°C unless otherwise indicated. The following strains were used in this study: N2 (var. Bristol) [54]; LGI: *zdIs5[mec-4::gfp]*; LGII: *vab-1*(*dx31*), *quIs5[mec-4::myr-vab-1]*; LG IV: *wsp-1(gm324)*, LG V: *unc-34(e566)*; LGX:, *quIs6[unc-34::unc-34::gfp]*; Unmapped: *quIs16[hs::myr-vab-1]* [55]; Extrachromosomal arrays (this study): *quEx131[mec-4::nck-1A]*, *quEx190[nck-1::nck-1A-gfp]* [12], *quEx215[mec-4::unc-34::gfp], quEx281*[*mec-4::unc-34*], *quEx283 [mec-4::nck-1A::gfp]* [12], *quEx321*[*mec-4::vca*], *quEx338[mec-4::unc-34 RNAi]* (see tissue specific RNAi). Unless noted otherwise, all *C. elegans* strains were obtained from the *C. elegans* Genetics Stock Center, (U. of Minnesota).

### Tissue-specific RNAi

To produce double stranded RNA (dsRNA) only in the mechanosensory neurons, we constructed a cloning vector (pIC659) with head to head P*mec-4* promoters on each side of a Multiple Cloning Site (MCS) such that the sense and antisense strands of an inserted cDNA would be transcribed. The *mec-4::unc-34 RNAi* construct (pIC727) was created by cloning an *unc-34* cDNA fragment (ATG start to the first SalI site, 388 bp) into the pIC659 dual P*mec-4 RNAi* cloning vector.

### Molecular biology

The *mec-4::nck-1A* construct (pIC313) was previously described in Mohamed and Chin-Sang (2011). The *mec-4::unc-34* construct (pIC624) was generated by amplifying *unc-34* cDNA and sub-cloning behind the *mec-4* promoter. The same procedure was used to make the *mec-4::unc-34::gfp* (pIC540) construct, but *unc*-34 was fused to *gfp* amplified from pPD95.75. To create the *mec-4::vca* construct (pIC673), the VCA region of WSP-1 (9108–9606 of the *wsp-1* gene; *C07G1.4a* in Wormbase) was amplified from genomic DNA and cloned behind the *mec-4* promoter. The *unc-34::unc-34::gfp* translation reporter was generated by a PCR fusion approach [56] using the following pieces: 1. A ∼5 kb genomic region that includes 2 kb of 5′UTR and the first two exons of *unc-34*, 2. Exons 2–7 were amplified from RB2 cDNA library, and 3. a 868 bp GFP fragment amplified from pPD95.75 (gift from Dr. Andrew Fire). The expression of the UNC-34::GFP rescued the *unc-34(e566)* uncoordinated phenotype. Details of plasmid/PCR constructs and primer sequences are available upon request.

### Transgenic animals

Transgenic animals were generated by germ-line transformation as previously described [57]. The *unc-34::unc-34::gfp* translational reporter was injected at a concentration of 20 ng/µL, and one of the *unc-34* rescuing lines (quEx61) was integrated to create *quIs6*. The *mec-4::unc-34* construct was injected at a concentration of 30 ng/µL into *mec-4::gfp(zdIs5); mec-4::myr-vab-1(quIs5)*. The *mec-4::unc-34::gfp* construct was injected at a concentration of 30 ng/µL into N2. *mec-4::unc-34RNAi, mec-4::vca* and *mec-4::nck-1* were injected into *mec-4::gfp(zdIs5)* at 30 ng/µL. *mec-4::nck-1(quEx131)* was later crossed into *unc-34(e566)*, and *mec-4::unc-34RNAi (quEx338)* was crossed into *vab-1(dx31)* and *wsp-1(gm324)*. Transgenic animals were identified by the co-injection marker pRF4/*rol-6* (30 ng/µl), or *odr-1::rfp* (30 ng/µl) [57]. At least two independent lines were isolated and analyzed. The data shown are from one representative line.

### Antibodies

Mixed stage animals were fixed and stained as described in Chin-Sang et al. (1999) [58]. Rabbit anti-VAB-1 antibodies (antigen VAB-1-HIS6) and chicken or mouse polyclonal antibodies against GFP (Chemicon) were used at 1∶100 dilutions. Texas Red-conjugated goat anti-rabbit and FITC conjugated goat anti-chicken or anti-mouse secondary anti bodies (Jackson's lab) were used at a 1∶100 dilution. For Western blot analysis, antibodies were used at the following dilutions: anti-NCK-1 at 1∶500, anti-VAB-1 at 1∶2500, anti-MBP-HRP at 1∶8000, anti-GST-HRP at 1∶4000 and 4G10 (Upstate Inc.) at 1∶2500. Goat-anti-rabbit-HRP and goat-anti-mouse-HRP were used as at 1∶10000 dilutions on western blots. Relative band intensities in Figure 6B were quantified using at least two independent blots and analyzed using the National Institutes of Health Image J program.

### Phenotypic analysis

The mechanosensory neurons were visualized using the *mec-4::gfp (zdIs5)* reporter. Young adult animals were scored as having PLM axon overextension or premature termination as described previously [6]. Outgrowth of the PLM axon happens during embryogenesis and continues to grow after hatching and most of its growth happens at the L1 stage. From L2 onwards to adulthood PLM growth is maintained relative to its termination point along the body [28]. To measure the L1 PLM axons, newly hatched L1s were synchronized in the absence of food for up to 12 hours. We found that although the worms were born in the absence of food that the PLM was still able to grow and the PLM axon lengths were equivalent to the length of animals developing for 2–3 hours post hatching. This corresponds to the Phase 1 or fast growth PLM growth phase [28]. Our wild-type reference strain *(zdIs5)* had L1 PLMs with an average PLM length of 108.5 (±5.5) microns with a PLM length/total body length (from head to tail) ratio of 0.48 (±0.04). L1 PLM axons were scored as overgrown if they were longer than 114 µm and had a PLM/total body length ratio of greater than 0.52. L1 PLM axons were scored as under grown if the PLMs were shorter than 103 µm and had a PLM length/total body length ratio of length less than 0.44. The L1 PLM axons were traced from photograph and measured in NIH Image J software. The wild-type neuron morphology was defined by analysis of neuronal GFP reporters and is consistent with the electron microscopic reconstruction of the *C. elegans* nervous system [59]. Animals were anesthetized using 0.2% tricaine and 0.02% tetramisole in M9, and mounted on 3% agarose pads. Unless stated otherwise, fluorescent animals and images were analyzed using a Zeiss Axioplan microscope, Axiocam and Axiovision software.

### Time-lapse imaging of PLM growth cones

PLM growth cones were visualized using a *mec-4::gfp (zdIs5)* reporter. Eggs were allowed to hatch for 5 minutes, and the newly hatched L1 animals were examined immediately on 3% agarose pads with a drop of 0.2% tricaine and 0.02% tetramisole in M9. PLM growth cones were imaged with a Zeiss LSM710 confocal microscope at intervals of 20–30 s. Axons were scored positive for filopodia if time-lapse movies revealed at least 2 protrusions, and there were dynamic movements (eg. growth and collapse) of the these structures within the 10–15 minutes of filming. See Videos S1 and S2 for examples.

### Yeast two-hybrid assays

Yeast cells were grown on standard and selective media as required [60]. The desired plasmids were transformed into yeast cells using the lithium acetate method [61]. For binding and deletion analysis, the pGBKT7 vector was used as bait and the pGADT7 vector (Clontech) as prey, and β-galactosidase activity was measured qualitatively by X-GAL overlay assays [62]. To identify interactions with VAB-1, the Kinase Region (669 aa-985 aa) of *vab-1* was cloned into pGBKT7 (pIC187) and used in a screen against the RB2 cDNA library (gift from Dr. R. Barstead), and about 600,000 colonies were screened and two independent *nck-1* cDNA clones were isolated. Site directed mutagenesis (QuickChange, Stratagene) of pIC187 was used to change the juxtamembrane tyrosine 673 changed to glutamic acid (Y673E). The SH2 domains of NCK-1, MIG-10, SEM-5, ABL-1 and VAV-1 were cloned into the activation domain of the pGADT7 vector. Primer sequences and details of plasmid constructs are available upon request.

### Pull down and co-purification assays

The following constructs were created by cloning the desired cDNA fragment into Glutathione-S-Transferase (pGEX4T-2, Amersham): pIC282 – NCK-1 SH2 domain (298 aa–397 aa), pIC297 – all three NCK-1 SH3 domains (1 aa–308 aa), pIC308 – 1^st^ NCK-1 SH3 domain (1 aa–72 aa), pIC593 – 2^nd^ NCK-1 SH3 domain (112 aa–186 aa), pIC309 – 3^rd^ NCK-1 SH3 domain (198 aa–308 aa), pIC324 – full length (F.L.) NCK-1 (1 aa–397 aa), and pIC606 – F.L. UNC-34 (1 aa–454). The following constructs were created by cloning the desired cDNA fragment into Maltose Binding Protein (pMAL^tm^-p2X, New England Biolabs): pIC225 – F. L. intracellular region of wild-type VAB-1 (581 aa–1117 aa), pIC119 – F. L. intracellular kinase deficient VAB-1 (G912E), pIC603 – UNC-34 RPO-EVH2 domain (128 aa–454 aa), pIC605 – F.L. UNC-34 (1 aa–454 aa), pIC671 – UNC-34 PRO domain (128 aa–274), pIC674 – UNC-34 EVH2 domain (246 aa–454 aa), pIC670 – WSP-1 VCA domain (334 aa–607 aa). pIC582 – His-6::VAB-1 (581 aa–1117 aa) was described in Brisbin et al (2009). All fusion constructs were expressed in *E. coli* Tuner (DE3). For Figure 4B, 4C, 4F, 4G and Figure 5A, a GST ‘pull-down’ assay was used to confirm the VAB-1, NCK-1 and UNC-34 interactions. Soluble/purified (Load) MBP-VAB-1, MBP-VAB-1(G912E), MBP-UNC-34 F.L., MBP-UNC-34-PRO-EVH2, MBP-UNC-34-PRO or MBP-UNC-34-EVH2 were incubated for 2–3 hrs at 4°C with soluble extracts containing either GST, GST-NCK-1 F.L., GST-NCK-1-all SH3 domains, GST-NCK-1(1^st^SH3), GST-NCK-1(2^nd^SH3), GST-NCK-1(3^rd^SH3), GST-NCK-1(SH2), His-6::VAB-1(581 aa–1117 aa) (pIC582) or GST-NCK-1 F.L. coexpressed with pIC582 bound to 50 µl glutathione sepharose beads (GE healthcare). Unbound fractions were collected, protein bound to GST beads were washed four times (25 mM Hepes, 10% Glycerol, 0.1% Triton-X, 285 mM NaCl), and a proportional loading of each sample was analyzed by standard SDS polyacrlyamide gel, followed by western blotting. All loads fused to MBP were detected using anti-MBP conjugated to HRP (New England Biolabs). His6-VAB-1 was detected using Rabbit anti-VAB-1 antibodies (antigen VAB-1-His6) (Figure 5A). GST and GST-NCK-1 F.L., and GST-NCK-1 deletion domains were detected either by Ponceau S or anti-GST conjugated to HRP. For Figure 6, MBP ‘pull-down’ was used to confirm VAB-1, NCK-1, WSP-1 and UNC-34 interactions. Soluble extracts (Load) of GST-NCK-1 F.L., His-VAB-1 (pIC582), GST-NCK-1 F. L. coexpressed with pIC582, or GST-UNC-34 F.L. were incubated for 2–3 hours at 4°C with soluble extracts containing either MBP or MBP-WSP-1(334 aa–608 aa) bound to 100 µl amylose resin beads (New England Biolabs). Unbound fractions were collected, protein bound to amylose beads were washed four times (20 mM Tris-Cl [pH 7.5], 200 mM NaCl, 1 mM EDTA, 1 mM DTT), and a proportional loading of each sample was analyzed by standard SDS polyacrylamide gel, followed by western blotting. VAB-1 was detected by Rabbit anti-VAB-1, GST fused proteins were detected by anti-GST conjugated to HRP, MBP and MBP-WSP were detected by anti-MBP conjugated to HRP.

## Supporting Information

Video S1 PLM growth cone of wild-type animals. Time-lapse confocal imaging of wild-type (N2) PLM growth cone. Images were taken at 30 s intervals for 15 minutes.
(MOV)

Video S2 PLM growth cone of hyperactive VAB-1 (*myr-vab-1*) transgenic animals. Time-lapse confocal imaging of *myr-vab-1* transgenic animals. Images were taken at 30 s intervals for 10 minutes.
(MOV)
